# Peripheral primitive neuroectodermal tumor causing cauda equina syndrome with destruction of L5 vertebra

**DOI:** 10.4103/0019-5413.65153

**Published:** 2010

**Authors:** Sarvdeep Dhatt, Mandeep S Dhillon, Sujit K Tripathy, Tarun Goyal, V Jagadeesh

**Affiliations:** Department of Orthopedics, Postgraduate Institute of Medical Education and Research, Sector 12, Chandigarh - 160 012, India; 1 Department of Orthopaedics, All India Institute of Medical Sciences, New Delhi, India

**Keywords:** Cauda equina syndrome, primitive neuro-ectodermal tumor, spinal tumor

## Abstract

A 24-year-old male patient presented with cauda equina lesion symptoms. His clinicoradiological examination including X-rays, CT scan and MRI revealed destruction of L_5_ vertebral body, pedicle and a mass extending to lateral recess and left intervertebral foramina causing pressure over the thecal sac. A CT guided FNAC was inconclusive. Open biopsy and hemilaminectomy of L_5_ vertebra was performed. Histopathology and immunocytochemical analysis revealed it to be primitive neuroectodermal tumor. Patient was given chemotherapy and radiation therapy. His lower limb power improved by grade I post operatively and at 2 years follow-up bowel/bladder recovery was noticed. Patient died after 2.5 years of surgery because of pulmonary metastasis.

## INTRODUCTION

Primitive Neuro-Ectodermal Tumor (PNET) is a small round cell tumor of neural crest origin, typically seen in second or third decades of life. These are highly malignant tumors, sharing the morphological, histochemical and immunogenetic features with Ewing sarcoma.^1^,^2^ It mainly exists in the central nervous system, chest wall, lower extremities, trunk, kidney and orbit but rarely in the spine. 3–8 Peripheral PNET (pPNET) of spine refers to all those tumors that arise from the surrounding soft tissues, vertebra or spinal nerve roots. To best of our knowledge, only 13 cases of PNET involving cauda equina have been reported in English language literature.^3^,^5^,^6^ This article discusses about the diagnostic and therapeutic complexities of PNET of L5 vertebra in a young patient who presented with cauda equina lesion symptoms.

## CASE REPORT

A 24-year-old male presented with a four-month history of progressive low back pain. He had fever, loss of appetite and significant loss of weight in the preceding four months and had already completed four months of antitubercular therapy as advised by a local physician who diagnosed the entity to be Pott’s spine of L5 vertebra. On clinical examination of the back we noticed a soft tissue swelling in the lumbar region measuring about 10 × 8 cm in dimensions. It was tender, warm; but nonmobile and nonpulsatile. On neurological examination he had, power around knee and ankle joint 3/5 grade motor, with diminished ankle and knee reflexes and bowel bladder incontinence.

Laboratory parameters revealed normal blood count with significantly raised erythrocyte sedimentation rate (ESR=68 mm/hr), alkaline phosphatase (ALP=528 U/L) and lactate dehydrogenase (LDH=355 U/L). Liver, Renal, Thyroid function and calcium profiles were all within normal range.

**Figure 1:**
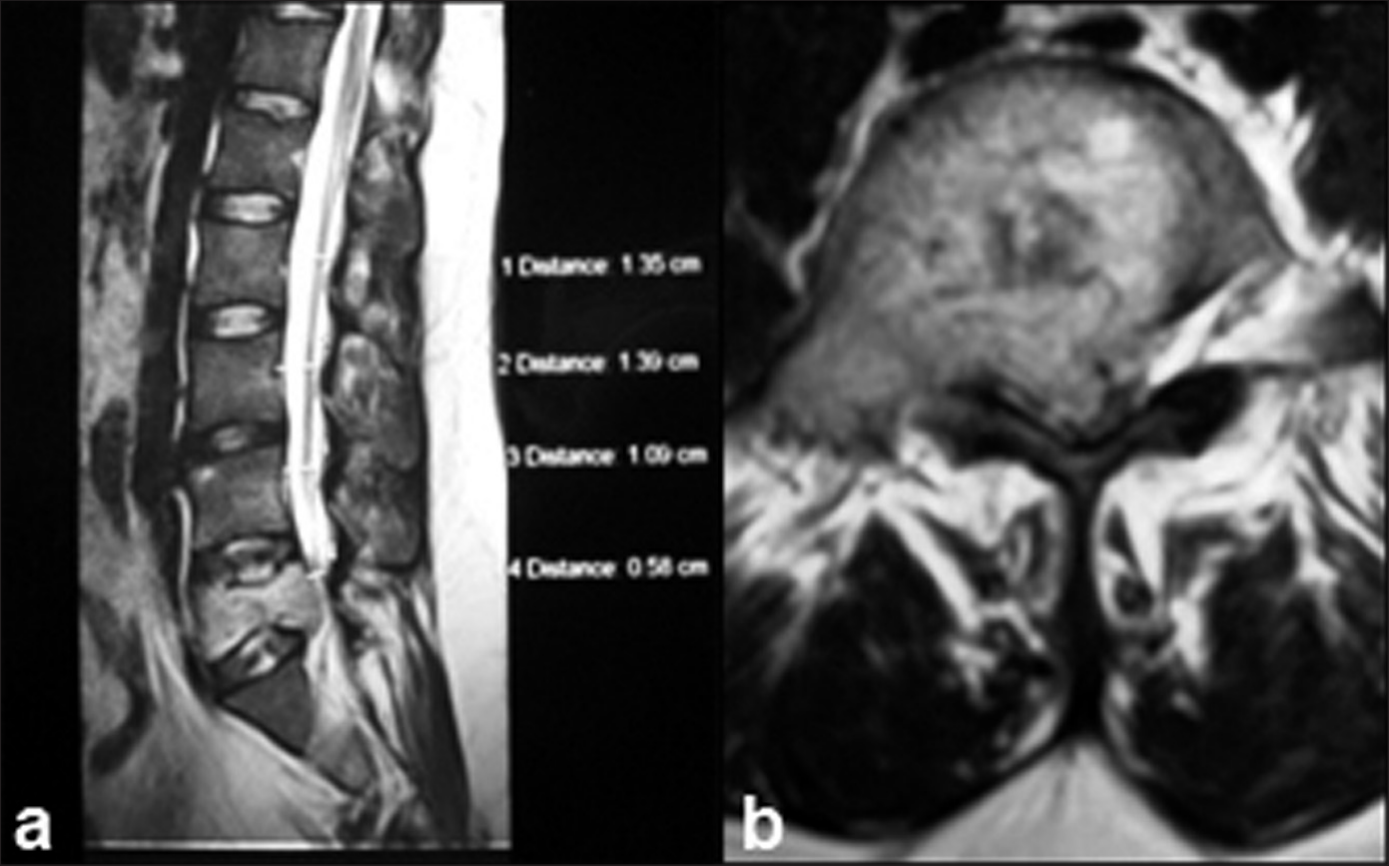
T_2_W MRI (a) saggital section showing cauda equina compression (b) Transverse section at L5 vertebral level showing complete involvement of the body, pedicles and extra spinal region

Radiograph and Computed tomography (CT) revealed marked destruction of L5 vertebra, extending to left pedicle and the left lateral elements. There was a left posterior extraosseous, paraspinal soft tissue component measuring 7 × 6 cm. On Magnetic Resonance Imaging (MRI), the mass had increased intensity on T2 and decreased intensity on T1 weighted images [Figure 1a]. The lesion extended to the lateral recesses and left intervertebral foramina, with significant thecal sac and cauda equina compromise [Figure 1b]. Anterior paravertebral extension was also seen with sparing disk spaces. These clinico-radiological pictures raised the possibility of malignancy. However CT guided FNAC was inconclusive with a hemorrhagic tap. Further investigation (Contrast enhanced CT of chest) showed a metastatic nodule in the lung. There was no other visceral involvement and no increased activity was noticed in any other skeleton on 3 phase MDP bone scan.

In view of progressive neurological deficit and inconclusive CT guided biopsy, open biopsy was planned with simultaneous extensive posterior decompression and stabilization. However, because of excessive bleeding from the tumor mass, the procedure could not be completed and was limited to left sided hemilaminectomy of L5 vertebra.

**Figure 2:**
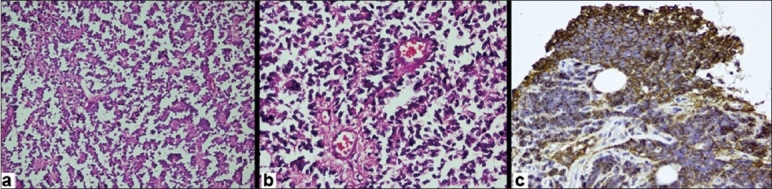
(a) Photomicrograph of histopathological specimen of the patient shows predominant round cells arranged in solid sheets or nests with hyper cellularity (magnification 20×10). (b) Magnified view of histology (40×10) shows small round cells forming true rossete. The cells show presence of hyperchromatic nuclei and scanty cytoplasm. (c) Immunocytochemistry demonstrated MIC-2 positivity (brown stained cells)

The microscopic picture of the biopsy specimen showed tumor cells comprised of small round cells arranged in solid sheets and nests with formations of true rosette. The tumor cells had hyperchromatic nuclei with scanty cytoplasm [Figure 2a, b]. A few areas of necrosis and hemorrhage were noticed, and it showed invasion into the adjacent soft tissue. Immunocytochemistry for MIC-2 and Neuron-specific enolase was found to be positive [Figure 2c]. The diagnosis of PNET involving the L5 vertebra was established.

After the surgical procedure, the patient had grade one power improvement on left side with mild subjective improvement in numbness; but the bowel-bladder incontinence persisted. MRI of the brain was found to be normal and hence the possibility of drop metastasis was excluded. He was further managed by radiation-oncologist and was administered multiagent chemotherapy (Vincristine, Adriamycin and Cyclophosphomide) and locally fractionated dose external beam radiotherapy (total 50 Gy). After a two-year follow-up, the patient had complete sensory and bowel-bladder recovery with motor power of 4/5 around ankle and foot. Six months later, he died of respiratory complications because of pulmonary metastasis.

## DISCUSSION

Irrespective of its origin (soft tissue, vertebra or spinal cord), the clinical picture, treatment and complications of spinal PNET remains same. Most of these cases present with history of progressive increase in back pain and neurological deficit. Radiography of vertebral PNET typically shows destructive lesion of the spine, sometimes associated with a soft tissue mass.^3^ Majority of the lesions are purely lytic, and most arise in posterior elements, extending to the vertebral bodies. Extension into the spinal canal is common, with progressive neurological deficit.

The lack of specific clinico-radiological features bring many primary differential diagnoses into consideration i.e.; Ewing sarcomas, osteosarcomas, neuroblastomas, lymphomas and leukaemias, chordomas, hemangoimas, aneurysmal bone cysts and giant cell tumors etc.^8^ Central type of vertebral tuberculosis with primary involvement and destruction of the vertebra is not uncommon in endemic area and is a strong diagnostic possibility. In fact, this patient was diagnosed as tuberculosis by the primary physician and empirically started on antitubercular drugs before he was referred to our tertiary care center.

Histological diagnosis in the form of fine needle aspiration cytology or open biopsy is essential to reach the diagnosis. PNET can be differentiated from other small round cell tumors, including lymphomas, by neural differentiation and immunocytochemical staining techniques.^1^,^2^,^9^,^10^ The present treatment of spinal PNET consists of local control in the form of surgical resection/ debulking surgery or radiotherapy, combined with systemic control with chemotherapy. 3–8,10 It is not possible to achieve complete surgical resection in the majority of the patients because of the extent of the tumor. Such patients must receive adjuvant radiotherapy. Peripheral PNETs can arise from peripheral nerves located within the intracranial or intraspinal leptomeninges (intradural) or from the nerve root after its exit from the neural foramina (extradural). The extradural tumor can arise from the surrounding soft tissue and vertebra also. Normal MRI of brain and spine in the present study excluded any primary pathology in the Central nervous system. Hence the possibility of ‘drop metastasis’ was excluded. The tumor arose from the L5 vertebra and caused compression of the thecal sac and cauda equina secondarily. These tumors are difficult to resect because of the nerve root involvement and carry an extremely poor prognosis with increase chances of recurrence and metastasis leading to dismal survival rates.^3^–^8^

Ozdemir^12^ reported that out of 10 cases of extradural tumor reported before, two died after a mean period of 18 months and seven were alive at 25 months. Survival of one patient was unknown. He pointed out that the follow up period was too short in these studies^12^. In his report, a patient with pPNET died after 14 months because of local recurrence after excision. Smorenberg *et al*, in their review of literature of 28 cases of spinal PNET found that 11 patients (39.3%) died at follow up period of 20 months.^2^ Similarly, Shi-sang He *et al*. reported the average time from operation to death was 18.1 months.^3^ The index case in this report died after 30 months of surgery because of pulmonary metastasis.

Though rare, PNET of spine is an important consideration in patients presenting with low back pain with soft tissue lump. Histology with immunocytochemical staining is essential for confirmation of diagnosis. Despite an aggressive management, the overall prognosis remains poor,^2^,^3^,^11^,^12^
